# Novel macrocycles: from synthesis to supramolecular function

**DOI:** 10.3762/bjoc.22.76

**Published:** 2026-06-24

**Authors:** Veronica Iuliano, Carmen Talotta, Margherita De Rosa, Paolo Della Sala, Konrad Tiefenbacher, Pablo Ballester, Carmine Gaeta

**Affiliations:** 1 Dipartimento di Chimica e Biologia “A. Zambelli”, Università di Salerno, Via Giovanni Paolo II 132, 84084 Fisciano, Salerno, Italy; 2 Department of Chemistry, University of Basel, Mattenstrasse 22, 4058 Basel, Switzerland; 3 Department of Biosystems Science and Engineering, ETH Zurich, Schanzenstrasse 44, 4056 Basel, Switzerland; 4 Institute of Chemical Research of Catalonia (ICIQ), Avgda. Països Catalans 16, 43007 Tarragona, Spain; 5 Catalan Institution for Research and Advanced Studies (ICREA), Passeig Lluís Companys 23, 08010 Barcelona, Spain

**Keywords:** macrocycles, supramolecular chemistry, supramolecular function

In supramolecular chemistry [1], macrocycles have long occupied a central position because of their ability to emulate the fundamental characteristics of biological receptors. Their well-defined cavities, reminiscent of enzymatic active sites, provide an ideal environment for multiple noncovalent interactions with guest molecules, thereby enhancing binding affinity and recognition selectivity [1–4].

Between 1980 and 2000, calixarenes [5–6], resorcinarenes [7], and cucurbiturils [8] emerged as some of the most extensively investigated classes of macrocyclic hosts. Their widespread popularity was driven by their straightforward functionalization and unique structural properties. In contrast to first-generation receptors, such as crown ethers [2], cryptands [3], and spherands [4], calixarenes and resorcinarenes feature aromatic cavities that accommodate a wide variety of neutral and charged organic guests.

A major advance in the field occurred in 2008, with the introduction of pillararenes by Ogoshi and co-workers [9]. Composed of 1,4-dialkoxybenzene units connected through methylene bridges, pillararenes are generally regarded as representatives of a third generation of macrocyclic hosts. Unlike the cone-shaped architecture typically adopted by calix[4]arenes, pillararenes display a rigid pillar-like geometry with a preorganized cavity. Their distinctive structural features and supramolecular behavior stimulated the development of numerous related macrocycles, including biphenarenes [10], oxatubarenes [11], saucerarenes [12], pagodarenes [13], calix[2]naphth[2]arenes [14], naphthotubes [15], and finally prismarenes [16], all of which are characterized by deep, biomimetic cavities capable of efficient guest encapsulation.

Macrocycles have evolved well beyond academic model studies and are now involved in a broad range of applications spanning environmental remediation [17], biomedicine [18–19], sensing [20–22], catalysis [23–25], and molecular separation [26–28]. Their practical relevance is further highlighted by their incorporation into commercial products and technologies, including the household deodorizer Febreze [29], novel cyclodextrin-based adsorbent DEXSORB [30], diagnostic devices [31], and drug-delivery platforms [32].

A major driving force in the field is the effort to combine synthetic versatility of macrocycles with practical supramolecular performance. This expanding structural diversity has, in turn, enabled increasingly sophisticated supramolecular functions. Precise functionalization strategies allow researchers to tune binding properties, guest inclusion kinetics, and physical behavior. As a result, modern macrocyclic host–guest systems now operate at the interface of materials science and chemical biology. Given the rapid development of this research area, we organized this thematic issue entitled “Novel macrocycles: from synthesis to supramolecular function”. The aim of this collection is to highlight recent advances in the design, synthesis, and functional applications of macrocyclic supramolecular receptors.

The issue opens with the work of Isaacs and co-workers [33], who report the synthesis of a new cucurbit[*n*]uril-like receptor functionalized with alkyl sulfate groups. The study compares the new host with a previously reported sulfonate-bearing analogue to evaluate how subtle modifications of the solubilizing ionic groups influence host–guest behavior. Although the introduction of sulfate groups decreases overall water solubility, it significantly enhances binding toward quaternary ammonium guests relative to primary ammonium ions. Detailed calorimetric and crystallographic data confirm that modifying the outer ionic environment is an effective tool to balance cation–dipole interactions against hydrophobic effects.

Complementing this focus on ionic interactions, the thematic issue proceeds with a study by Qi-Qiang Wang, De-Xian Wang, and coworkers [34] that addresses the long-standing challenge of recognizing organic dicarboxylate anions in solution. Using a one-pot synthetic strategy based on oxacalix[2]arene[2]triazine macrocyclic units, the authors prepared a family of functionalized ultracycle hosts. Encapsulation of flexible dicarboxylate guests arises from the combined action of hydrogen bonding and anion–π interactions, the latter of which is enhanced by the electron-deficient triazine rings. Importantly, the study demonstrates a pronounced size-matching effect, showing how cavity dimensions can be tuned to selectively recognize specific dicarboxylate chain lengths.

Subsequently, Kato, Ogoshi, and colleagues [35] report the selective monoformylation of naphthalene-fused propellanes. The observed regioselectivity originates from through-space electronic communication between the naphthalene units, which suppresses multiple substitution pathways across the remaining framework. Owing to their defined three-dimensional shape and moderate conformational flexibility, the resulting desymmetrized building blocks avoid the dense packing commonly observed in rigid planar systems. As a proof of concept, these precursors were converted into methylene-alternating copolymers that exhibit high solubility and measurable carbon dioxide adsorption, highlighting their potential for the development of functional soft materials.

Asymmetric frameworks are also central to the research reported by Niemeyer and co-workers [36], who focused on the synthesis of crown ethers bearing one or two BINOL units. Their paper outlines highly practical synthetic routes to open a diverse library of chiral architectures with modular ring sizes and peripheral groups. Incorporating either one or two BINOL units, these molecules represent highly versatile, nonsymmetric building blocks as promising candidates for further application in enantioselective chemosensing or organocatalysis.

Providing a comprehensive biomedical perspective, Yang, Wang, Yao, and co-workers [37] discuss recent progress in smart supramolecular drug delivery systems. Their Review article highlights the utility of calixarenes and pillararenes as platforms for controlled-release applications due to their hydrophobic cavities, straightforward functionalization, and selective host–guest recognition properties. By organizing recent examples by internal and external stimuli, the authors outline how macrocyclic systems can regulate cargo release via supramolecular recognition and nanovalve mechanisms. The Review also discusses current challenges and future directions for the clinical translation of these materials.

Moving from broad biomedical overviews to therapeutic applications, Consoli and co-workers [38] exploit the synthetic versatility of calix[4]arenes to develop selective anticancer systems. By clustering bioactive isothiouronium groups on an amphiphilic calixarene scaffold bearing long alkyl chains, the authors obtained a derivative that spontaneously self-assembles into stable nanoscale aggregates in water. Biological studies revealed a potent antiproliferative effect against human renal carcinoma cells, with limited toxicity toward healthy cells. In addition to its intrinsic biological activity, the nanoassembled platform may also serve as a carrier for combination therapies by encapsulating additional therapeutic agents.

Macrocyclic chemistry is proving equally vital for environmental sustainability and separation science. For instance, Šindelář and co-worker [39] designed a functionalized silica gel embedded with covalently immobilized bambusuril macrocycles. This material was engineered for the selective sorption of dicyanoaurate(I), an anion highly relevant to gold mining waste streams. The solid-supported system retains its high extraction efficiency even when flooded with competing anions. Furthermore, by evaluating the recyclability and solvent stability of covalent versus noncovalent immobilization, the study offers key design rules for building durable separation materials.

While solid-supported systems demonstrate the application potential of macrocyclic chemistry, their further development relies on access to versatile and selectively functionalizable scaffolds. In this context, Vatsouro and co-workers [40] report efficient methodologies for accessing heteromultifunctional calix[4]arene derivatives. Through carefully designed protection and functionalization sequences, the authors introduced orthogonal reactive groups at opposite rims of the macrocycle. The synthetic versatility of these systems was demonstrated by copper-catalyzed “click” reactions and amine-based transformations, yielding highly functionalized derivatives that assemble into homo- and heterodimeric supramolecular capsules. This work establishes adaptable synthetic platforms for the preparation of increasingly complex supramolecular architectures.

The development of such multifunctional systems can also help address major environmental challenges. Danjou and colleagues [41] explored the use of macrocyclic scaffolds for environmental remediation, particularly in the extraction of radioactive contaminants. The authors synthesized a phenoxycalix[4]pyrrole framework functionalized with multiple hydroxamic acid groups for the selective removal of uranyl(VI) ions from acidic aqueous media. Solid–liquid extraction experiments demonstrated efficient sequestration of the targeted species, highlighting the potential of preorganized macrocyclic receptors in the development of high-performance materials for nuclear waste treatment.

A novel class of peptide-functionalized supramolecular tweezers was developed by Schrader and Vetter [42] to target survivin, a key component of the chromosomal passenger complex (CPC). These receptors were designed to interfere with the recognition of phosphorylated histone H3 by the survivin BIR domain, displaying high binding affinity and selectivity that depended on the presence of Lys121. Structural investigations revealed an unexpected binding mode in which, in addition to the peptide-mediated recognition of the BIR domain, the tweezer scaffold established supplementary interactions with the protein surface. These findings provided valuable insights into the molecular basis of survivin recognition and guided the design of second-generation peptide tweezers capable of simultaneously engaging multiple binding regions of the protein. The study highlights the potential of peptide-modified supramolecular tweezers as versatile tools for the selective modulation of biologically relevant protein–protein interactions.

The contributions collected in this thematic issue highlight the breadth and continued evolution of contemporary macrocyclic chemistry. From new ultracycle receptors to functional materials for carbon dioxide adsorption, separation science, environmental remediation, and biomedical applications, these studies demonstrate how advances in synthetic design continue to expand supramolecular function. As macrocyclic systems become increasingly integrated into materials science and chemical biology, their impact across multiple research areas will continue to grow.

We sincerely thank all authors who contributed their work to this thematic issue. We are also grateful to the referees for their careful evaluations and valuable suggestions. Finally, we thank the Editorial Team of the *Beilstein Journal of Organic Chemistry* for their professional support.

Pablo Ballester, Konrad Tiefenbacher, Carmine Gaeta, Carmen Talotta, Margherita De Rosa, Paolo Della Sala

Tarragona, Basel, Salerno, June 2026

## Data Availability

Data sharing is not applicable as no new data was generated or analyzed in this study.

## References

[1] Lehn J-M (1995). Supramolecular Chemistry: Concepts and Perspectives.

[2] Pedersen C J (1988). Angew Chem, Int Ed Engl.

[3] Lehn J-M (1988). Angew Chem, Int Ed Engl.

[4] Cram D J (1988). Angew Chem, Int Ed Engl.

[5] Neri P, Sessler J L, Wang M-X (2016). Calixarenes and Beyond.

[6] Steed J W, Atwood J L (2013). Supramolecular Chemistry.

[7] Tunstad L M, Tucker J A, Dalcanale E, Weiser J, Bryant J A, Sherman J C, Helgeson R C, Knobler C B, Cram D J (1989). J Org Chem.

[8] Lagona J, Mukhopadhyay P, Chakrabarti S, Isaacs L (2005). Angew Chem, Int Ed.

[9] Ogoshi T, Kanai S, Fujinami S, Yamagishi T-a, Nakamoto Y (2008). J Am Chem Soc.

[10] Chen H, Fan J, Hu X, Ma J, Wang S, Li J, Yu Y, Jia X, Li C (2015). Chem Sci.

[11] Jia F, He Z, Yang L-P, Pan Z-S, Yi M, Jiang R-W, Jiang W (2015). Chem Sci.

[12] Li J, Zhou H-Y, Han Y, Chen C-F (2021). Angew Chem, Int Ed.

[13] Han X-N, Han Y, Chen C-F (2020). J Am Chem Soc.

[14] Del Regno R, Della Sala P, Spinella A, Talotta C, Iannone D, Geremia S, Hickey N, Neri P, Gaeta C (2020). Org Lett.

[15] Yang L-P, Wang X, Yao H, Jiang W (2020). Acc Chem Res.

[16] Della Sala P, Del Regno R, Talotta C, Capobianco A, Hickey N, Geremia S, De Rosa M, Spinella A, Soriente A, Neri P (2020). J Am Chem Soc.

[17] Skorjanc T, Shetty D, Trabolsi A (2021). Chem.

[18] Pan Y-C, Li J-X, Guo D-S (2025). ACS Cent Sci.

[19] Bier D, Rose R, Bravo-Rodriguez K, Bartel M, Ramirez-Anguita J M, Dutt S, Wilch C, Klärner F-G, Sanchez-Garcia E, Schrader T (2013). Nat Chem.

[20] Pinalli R, Pedrini A, Dalcanale E (2018). Chem Soc Rev.

[21] Tay H M, Beer P (2021). Org Biomol Chem.

[22] Minami T, Esipenko N A, Akdeniz A, Zhang B, Isaacs L, Anzenbacher P (2013). J Am Chem Soc.

[23] Raynal M, Ballester P, Vidal-Ferran A, van Leeuwen P W N M (2014). Chem Soc Rev.

[24] Zhang Q, Catti L, Tiefenbacher K (2018). Acc Chem Res.

[25] Ning R, Wang Q-Q (2025). Chem Soc Rev.

[26] Zhu H, Chen L, Sun B, Wang M, Li H, Stoddart J F, Huang F (2023). Nat Rev Chem.

[27] Ogoshi T, Sueto R, Yoshikoshi K, Sakata Y, Akine S, Yamagishi T-a (2015). Angew Chem, Int Ed.

[28] Ohtani S, Onishi K, Wada K, Hirohata T, Inagi S, Pirillo J, Hijikata Y, Mizuno M, Kato K, Ogoshi T (2024). Adv Funct Mater.

[29] (2026). https://www.febreze.com/en-us/ingredients-safety/our-ingredients.

[30] (2026). https://cyclopure.com/dexsorb/.

[31] Davis A P (2020). Chem Soc Rev.

[32] Rajewski R A, Stella V J (1996). J Pharm Sci.

[33] Akakpo C, Zavalij P Y, Isaacs L (2025). Beilstein J Org Chem.

[34] Mi W-H, Huang T-Y, Wang X-D, Ao Y-F, Wang Q-Q, Wang D-X (2025). Beilstein J Org Chem.

[35] Kato K, Hiroi T, Okada S, Ohtani S, Ogoshi T (2025). Beilstein J Org Chem.

[36] Kheirjou S, Riebe J, Thiele M, Wölper C, Niemeyer J (2025). Beilstein J Org Chem.

[37] Yi L-H, Qin J, Lu S-R, Yang L-P, Wang L-L, Yao H (2025). Beilstein J Org Chem.

[38] Granata G, Ferreri L, Leotta C G, Pitari G M, Consoli G M L (2025). Beilstein J Org Chem.

[39] Šusterová M, Šindelář V (2025). Beilstein J Org Chem.

[40] Korniltsev I, Bazhenov V, Gorbunov A, Cheshkov D, Bezzubov S, Kovalev V, Vatsouro I (2026). Beilstein J Org Chem.

[41] Karnib S, Baydoun R, Zaidan W, AlHaddad N, El Samad O, Nsouli B, Cazier-Dennin F, Danjou P-E (2026). Beilstein J Org Chem.

[42] Gsell C, Rebmann P, Opara K, Beuck C, Bayer P, Bier D, Vetter I R, Schrader T (2026). Beilstein J Org Chem.

